# Successful thoracic endovascular aortic repair for post-coarctoplasty aneurysm

**DOI:** 10.1186/s43044-020-00051-7

**Published:** 2020-03-30

**Authors:** Sedigheh Saedi, Maryam Aliramezany, Jamal Moosavi, Tahereh Saedi

**Affiliations:** 1 Rajaei Cardiovascular Medical and Research Center, Iran University of Medical Sciences, Vali-asr Ave, adjacent to Mellat Park, Tehran, Iran; 2 Afzalipour Hospital, Kerman University of Medical Sciences, Kerman, Iran

**Keywords:** Aorta, Coarctation, Aneurysm, Endovascular repair, Case report

## Abstract

**Background:**

Aortic coarctation is currently treated by both surgical and transcatheter methods. Patients can present with late complication of prior surgical repair including recoarctation and aneurysm formation. There are limited reports on safety and efficacy of thoracic endovascular aortic repair methods (TEVAR) in post-coarctation repair patients.

**Case presentation:**

We report an adult patient with aortic aneurysm formation following surgical coarctoplasty successfully treated with transcatheter TEVAR method obviating the need for open heart surgery.

**Conclusion:**

Endovascular repair of aneurysms in post-coarctoplasty patients is a promising method and should be considered in those with suitable anatomy based on prior imaging.

## Background

Aortic coarctation refers to the congenitally stenotic aorta, more commonly found near the ligamentum arteriosum and adjacent to the left subclavian artery [1]. Coarctation of the aorta occurs in one out of 2500 live births with a male to female ratio of 2:1 and is now treated either surgically or percutaneously. Coarctation repair can improve the survival and prognosis if the intervention is performed in time [2]. However, there are well-known entities in the follow-up of these patients including recoarctation and formation of true or false aneurysms. Here we report a patient with postoperative complication treated successfully by catheter intervention.

## Case presentation

**Fig. 1:**
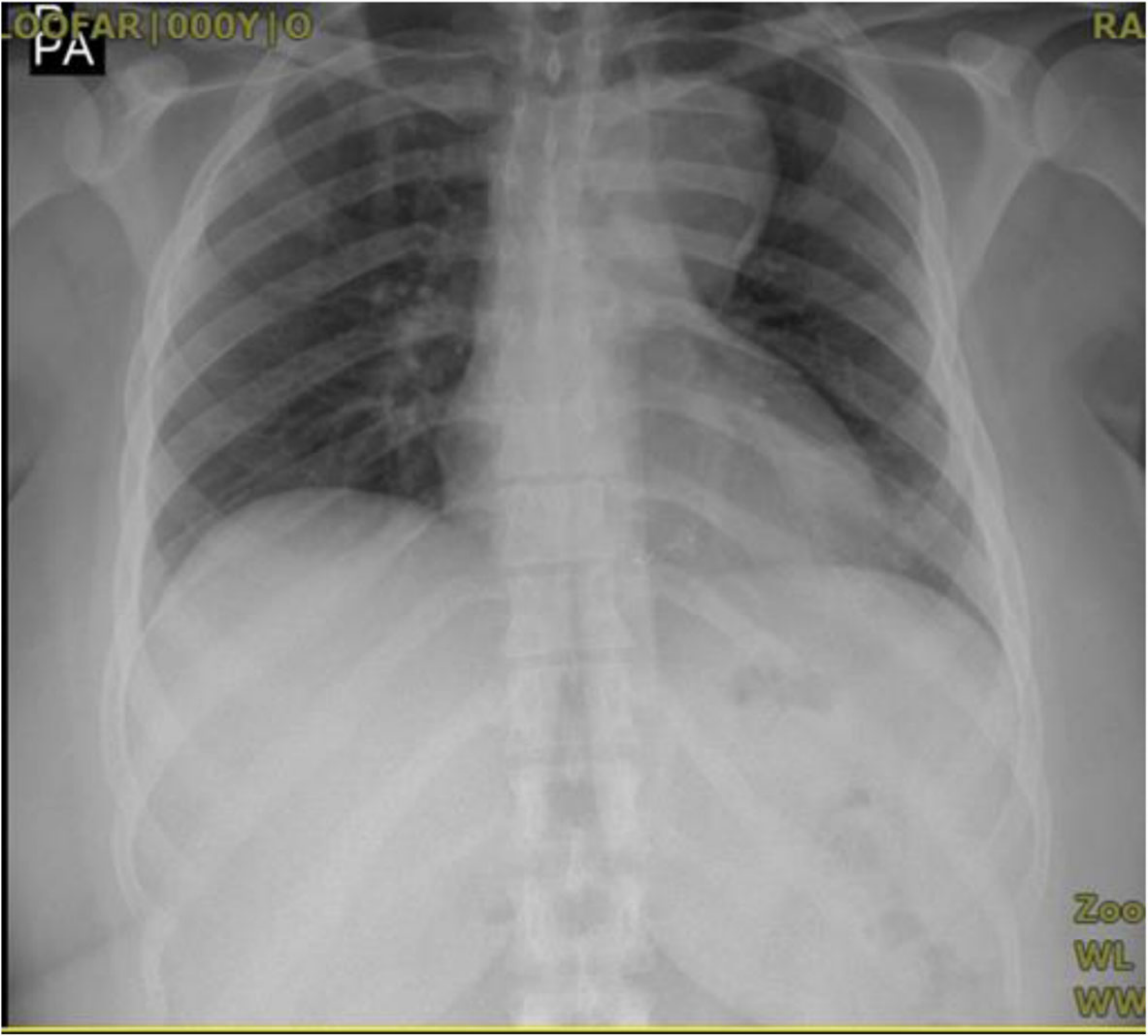
CXR depicting aneurysmal dilation of the aorta

**Fig. 2:**
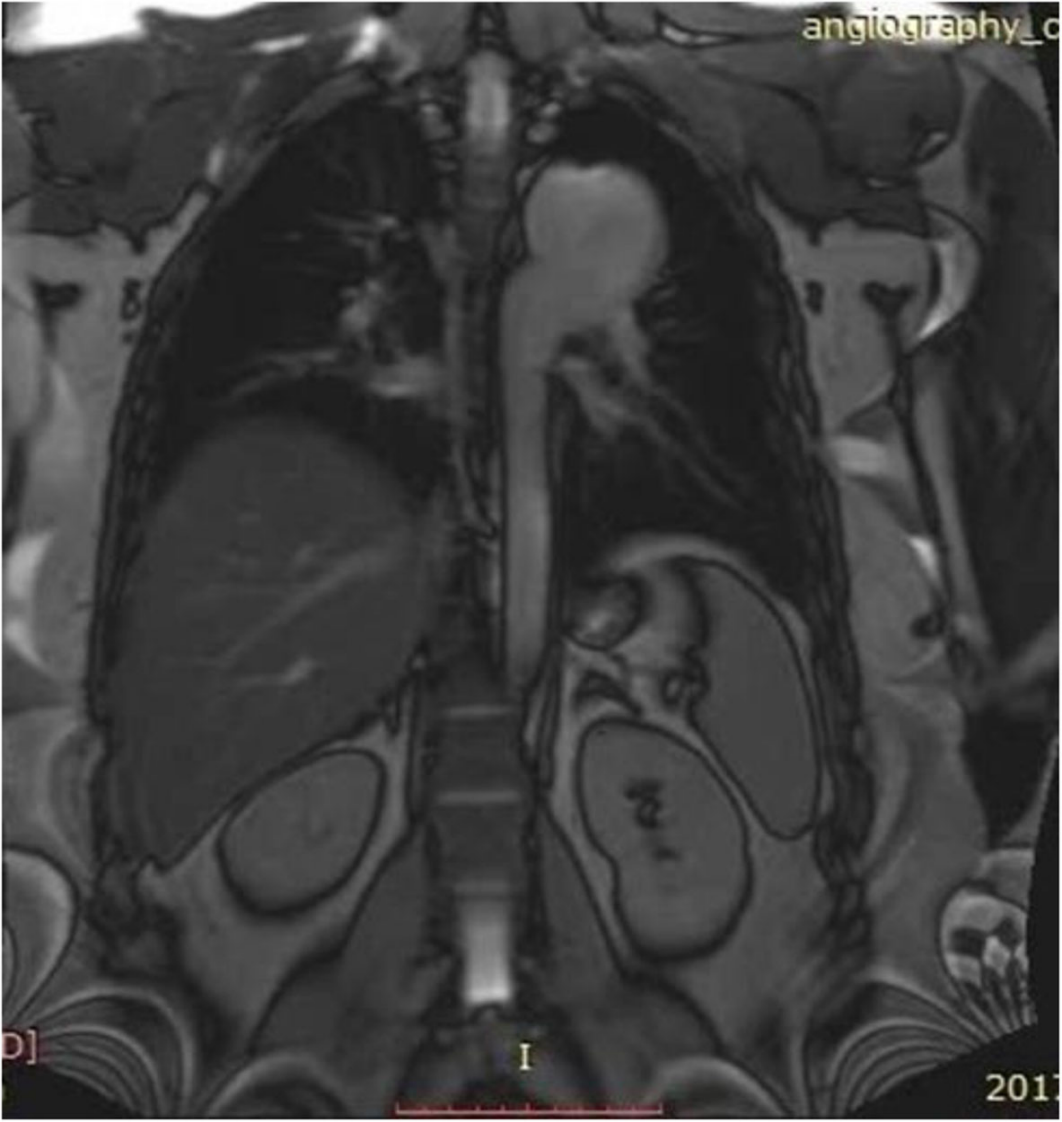
Cardiac magnetic resonance images showing aneurysmal dilation in distal part of aortic arch and proximal part of descending aorta (maximum diameter of 50 mm)

A 25-year-old female with history of surgical repair of coarctation in infancy and percutaneous device closure of a mid-muscular ventricular septal defect (VSD) at age eighteen presented to our adult congenital clinic for routine follow-up. She stated that she was asymptomatic and had normal functional capacity. Physical examination findings were within normal limits. Chest X-ray showed normal heart size and an abnormal bulging in the upper left heart border suggesting aneurysmal dilation of thoracic aorta (Fig. 1). Cardiac magnetic resonance (CMR) imaging was performed for surveillance of the coarctation repair site and revealed formation of a large aneurysm in distal part of the aortic arch and proximal part of descending thoracic aorta measuring an anteroposterior diameter of 50 mm that also involved the origin of the left subclavian artery (Fig. 2). Given the inherent risk of complications or rupture as the aneurysm had formed at site of previous surgical repair, we decided to have a lower threshold for intervention in this young patient. The patient refused any surgical options; therefore, percutaneous repair of the aneurysm by thoracic endovascular aortic repair (TEVAR) method was planned.

## Intervention

**Fig. 3:**
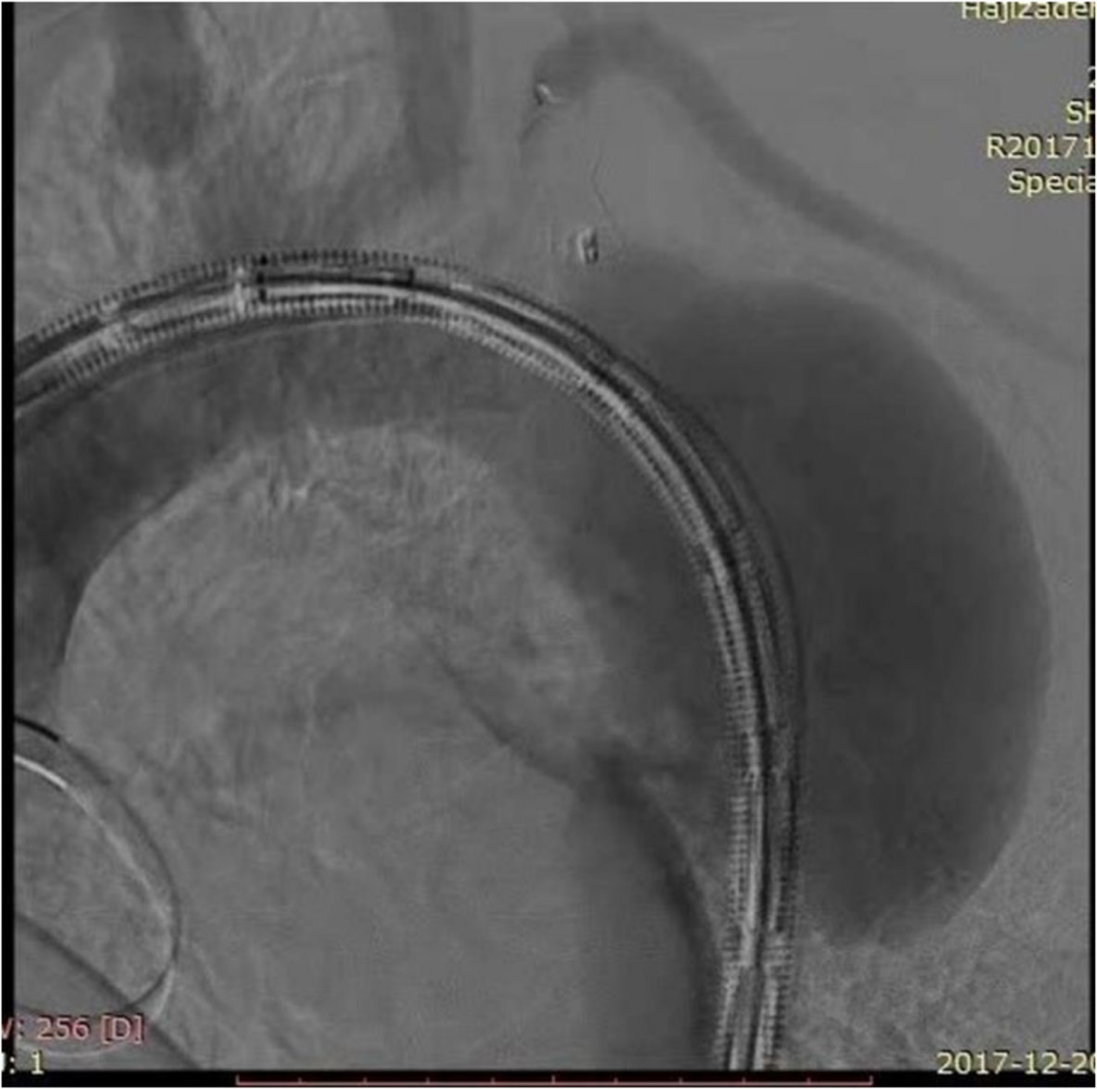
Aortic angiography showing the aortic aneurysm

Aortography was performed via femoral route, and injections were done using a radiopaque marker pigtail to get accurate measurements. Aortic angiography confirmed CMR findings (Fig. 3).

**Fig. 4:**
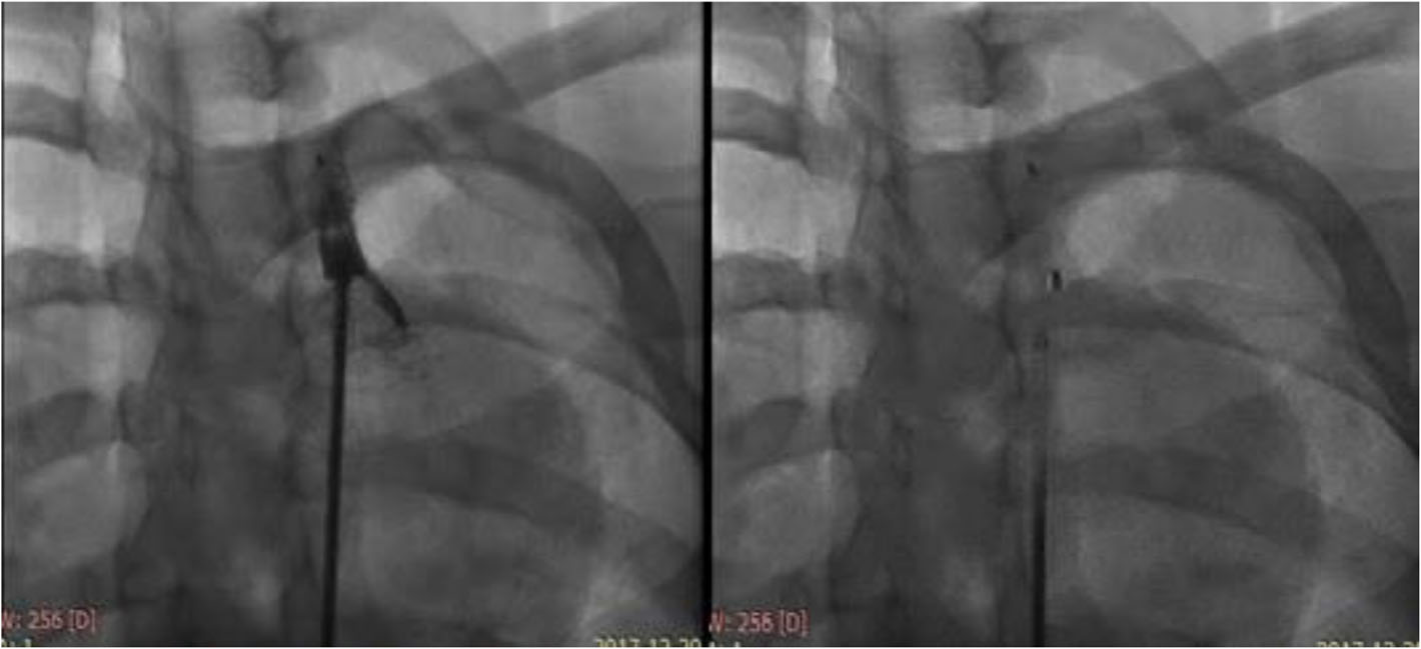
Obstruction of the subclavian artery with PDA device

**Fig. 5:**
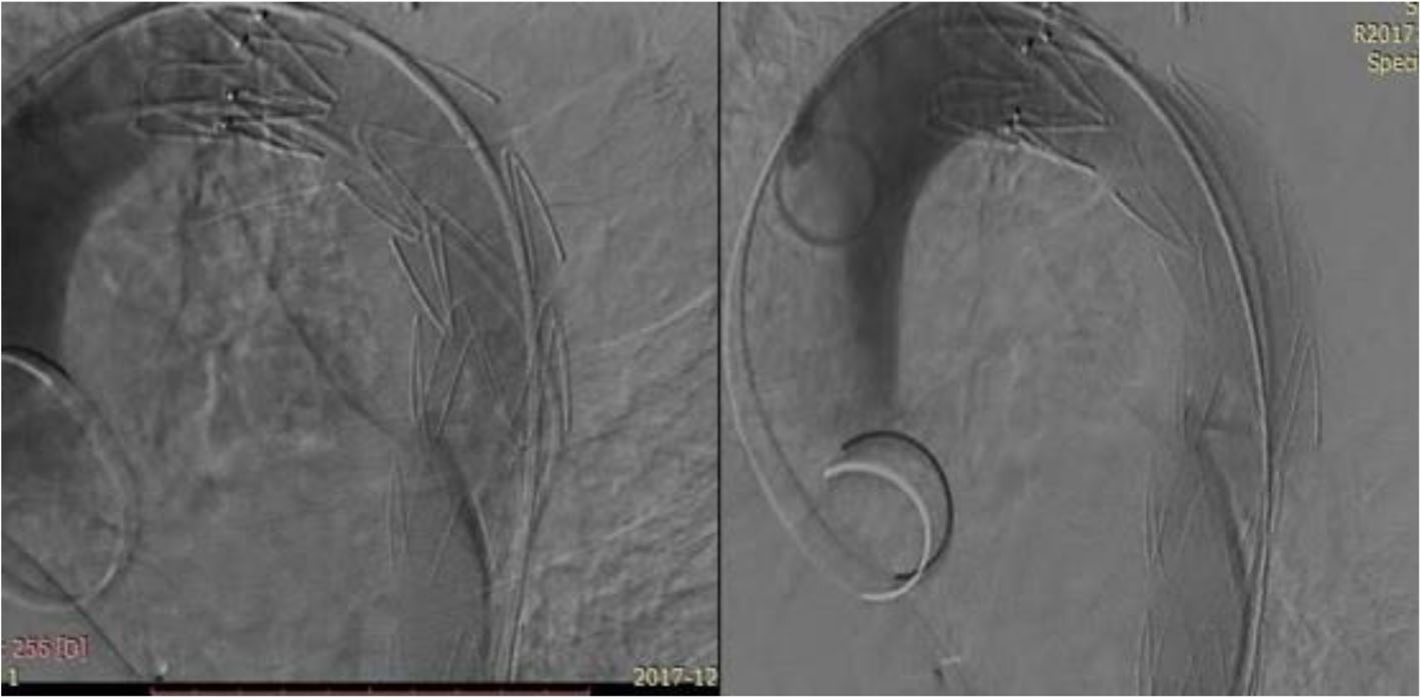
Stent graft deployed just after left carotid artery

Initially, the left subclavian artery ostium was occluded using a Occlutech® PDA occluder 8 × 10 mm to eliminate the risk of endoleaks (Fig. 4).Then a 24 × 24 × 120 mm stent graft (Zenith Alpha™ Thoracic Endovascular Stent Graft ,Cook Medical) was deployed just after left carotid artery with successful results and without complication (Fig. 5). Stent length was selected based on pre-procedural CT angiography, and the stent was costume made to be 20% longer than the CT-measured diseased portion of the aorta of about 93 mm.

**Fig. 6:**
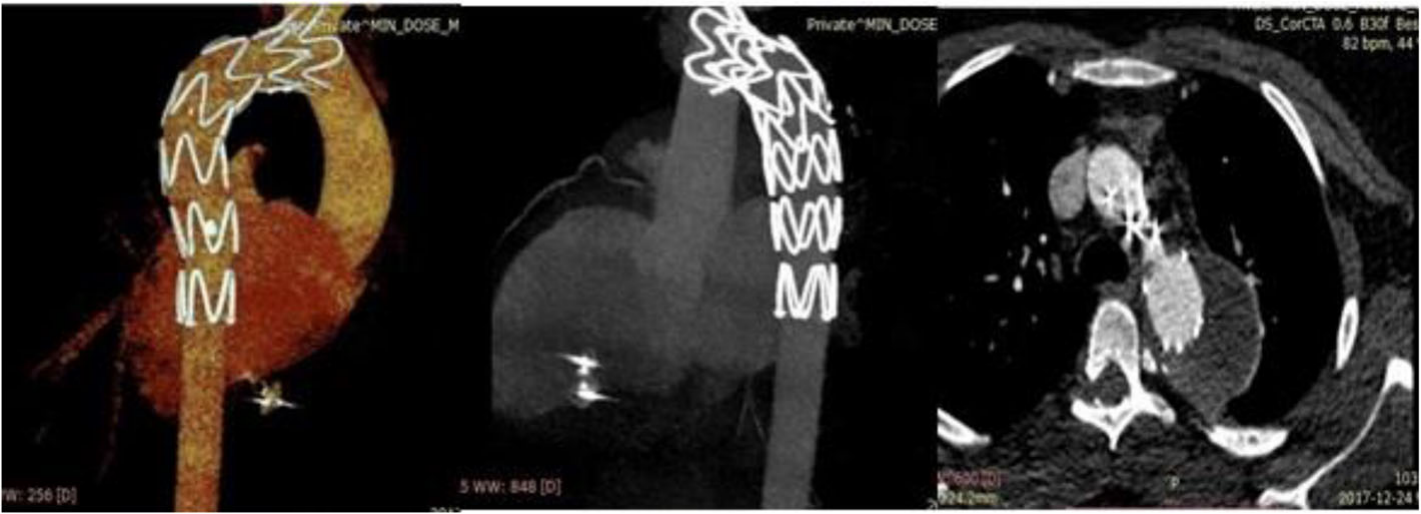
Follow-up CT angiography showing complete exclusion of the aneurysm

Follow-up CT angiography showed complete exclusion of the aneurysm with no complications (Fig. 6). At 1-year follow-up post-intervention, the patient was doing well with no reported worrisome issues.

## Discussion

Repair of aortic coarctation has traditionally been based on surgical methods although catheter interventions are progressively improving [3]. Different surgical approaches including resection with end-to-end anastomosis, direct aortoplasty using transverse suture repair, patch-graft aortoplasty, subclavian flap aortoplasty, and resection with end-to-end conduit interposition are used for surgical correction. In spite of successful repair of coarctation, postsurgical patients are still at risk of long-term complications including recurrence of the aortic coarctation and true or false aortic aneurysm formation with risk of dissection, rupture, or fistulization to adjacent structures. Although aneurysm formation at the site of repair is relatively more common after patch graft aortoplasty it is seen following other approaches including bypass grafting, end-to-end anastomosis, or subclavian flap aortoplasty and even after transcatheter interventions [4]. The aneurysm usually develops as a result of a more flexible vessel wall compared to the patch, long-lasting hypertension, or extreme resections of the aortic rim [3]. Hence, to protect patients against complicated aneurysms, we need to identify them early by regular follow-up imaging. Relatively high mortality and morbidity is seen with the repeat surgical repair of postsurgical complications, and the best management strategy has yet to be established .Reoperation following previous patch-graft aortoplasty is reported to have a 14% mortality rate and substantial morbidity, including paraplegia and bleeding complications [5]. In addition, patients often prefer minimally invasive options.

Although endovascular stent grafting has been approached cautiously in the treatment of such young patients, newer stent graft designs offer better conformability and durability, and endovascular stent grafting could be considered as a safe option for treatment of these patients. Advantages of stent grafts include the fact that they could cover the total length of the diseased aorta. However, long-term efficacy and freedom of reintervention remain to be investigated.

Yazar et al. in their series of 13 patients with TEVAR for treatment of late complications after aortic coarctation reported cases suffering mortality and morbidity [6]. Lala et al. reported 21 adult patients with primary coarctation or post repair complications, four of whom had pseudoaneurysm and were treated with TEVAR, with acceptable results and no mortality. However, there were cases with reported endoleaks and patients requiring reinterventions during their 8 months of follow-up [7]. Erben et al. also reported 11 patients with postoperative aneurysm/pseudoaneurysm in their case series, all of whom were treated with stent grafts. Four patients needed concomitant left carotid to left subclavian artery bypass and two a right carotid to left carotid to left subclavian artery bypass [8]. Our patient had the left subclavian artery prophylactically occluded to avoid the risk of endoleak. As the patient did not develop upper extremity claudication, there was no need for left subclavian bypass grafting.

## Conclusion

The present case highlights the importance of considering percutaneous methods of aneurysm repair in patients who are optimal candidates based on prior imaging, over the routine open surgical alternatives and thereby averting the risks associated with repeat surgical procedures. Large and multi-center trials with long-term follow-up focusing on postsurgical patients are needed to further clarify the role and potential shortcomings of the endovascular stent grafts in the management of these patients.

## Data Availability

N/A

## Abbreviations

VSD: Ventricular septal defect
PDA: Patent ducus arteriousus
